# Evaluating the Gaps in the Diagnosis and Treatment in Extra-Pulmonary Tuberculosis Patients Under National Tuberculosis Elimination Programme (NTEP) Guidelines: A Multicentric Cohort Study

**DOI:** 10.3390/tropicalmed10080206

**Published:** 2025-07-24

**Authors:** Sanjeev Sinha, Renuka Titiyal, Prasanta R. Mohapatra, Rajesh K. Palvai, Itishree Kar, Baijayantimala Mishra, Anuj Ajayababu, Akanksha Sinha, Sourin Bhuniya, Shivam Pandey

**Affiliations:** 1 Department of Medicine, All India Institute of Medical Sciences, Ansari Nagar, New Delhi 110029, India; titiyalrenuka@gmail.com (R.T.); anuj.ajaybabu@gmail.com (A.A.); akankshasinha2912@gmail.com (A.S.); 2 Department of Pulmonary Medicine & Critical Care, AII India Institute of Medical Sciences, Bhubaneswar 751019, India; prmohapatra@hotmail.com (P.R.M.); sbhuniya@hotmail.com (S.B.); 3 Government General & Chest Hospital, Hyderabad 500013, India; dr.rajpalvai@gmail.com; 4 Department of Biostatistics, All India Institute of Medical Sciences, Ansari Nagar, New Delhi 110029, India; dr.shivampandey@yahoo.com

**Keywords:** extra-pulmonary tuberculosis (EPTB), National Tuberculosis Elimination Programme (NTEP), Xpert MTB/Rif, patient delay, health system diagnosis delay, total treatment delay

## Abstract

Extra-pulmonary tuberculosis (EPTB) can affect any organ of the body, producing a wide variety of clinical manifestations that make the diagnosis and treatment of EPTB challenging. The optimum treatment varies depending on the site of EPTB, its severity, and response to treatment. There is often uncertainty about the best management practices, with a significant departure from national guidelines. This study aims to identify gaps and barriers in adhering to the national guidelines for the diagnosis and treatment of EPTB. We included 433 patients having EPTB and followed up at predefined intervals of 2 months, 6 months, 9 months, and 12 months. Questionnaire-based interviews of the treating physician and the patients in different departments were conducted. For confirmatory diagnosis, heavy dependence on clinical-radiological diagnosis without microbiological support was observed, which is a deviation from National Tuberculosis Elimination Programme (NTEP) guidelines and raises concerns about the potential for misdiagnosis and overtreatment. Apart from patient delays, long health system delays in EPTB were observed. The median patient delay, health system delay, and total treatment delay times were 4.2, 4, and 10.1 weeks, respectively. To enhance EPTB diagnosis and management, there is a pressing need for improved access to microbiological testing, enhanced physician training on adherence to NTEP guidelines, and greater utilisation of imaging and histopathological techniques.

## 1. Introduction

Extra-pulmonary tuberculosis (EPTB) remains a substantial component of the global tuberculosis (TB) burden, especially in lower socioeconomic countries. According to the World Health Organization (WHO), an estimated 10.8 million TB cases were reported globally in 2024 [1]. In India, EPTB constituted approximately 20–24% of total TB cases across all age groups, with a notably higher prevalence observed among immunocompromised individuals than in the general population [2]. Although considerable advancements have been made in the diagnosis of pulmonary TB, confirmatory diagnosis in EPTB remains a diagnostic challenge. Data from the Nikshay platform indicate that India recorded over 2.4 million TB cases in 2022, with EPTB accounting for 24% of these. Notably, the proportion rises to more than 50% among individuals co-infected with HIV [3]. Despite improvements in diagnostic tools and the expansion of TB-related healthcare services, delays in EPTB diagnosis persist, primarily due to limited health-seeking behaviour among patients and the complexity of confirming EPTB through microbiological methods [4]. Confirming the microbial diagnosis of EPTB is often difficult, leading to diagnostic delay. Research on pulmonary TB has shown that patient-related delays contribute to nearly 77% of the total time before treatment initiation [5,6]. Under the NTEP, public awareness campaigns primarily focus on the hallmark symptoms of pulmonary TB, which may leave patients unaware of EPTB manifestations. Previous observations suggest that many EPTB patients present with advanced disease stages at the time of diagnosis. Thus, assessing patients’ awareness and their health-seeking behaviour is crucial to formulating effective strategies for timely diagnosis and management of EPTB in India [7,8]. There is often uncertainty about the best management practices, with a significant departure from NTEP guidelines. In this study, we aimed to identify the gaps and barriers in adhering to the national guidelines for the diagnosis and treatment of EPTB. This approach aimed to assess diagnostic and management practices and identify areas for potential improvement in the implementation of existing guidelines.

## 2. Materials and Methods

### 2.1. Study Design

This study was a prospective cohort study conducted across three centres in India, namely, All India Institute of Medical Sciences (AIIMS), New Delhi; AII India Institute of Medical Sciences, Bhubaneswar; and Government General & Chest Hospital, Hyderabad.

### 2.2. Participants

Participants were recruited from 1st March 2023 to 28 February 2025 from inpatients and outpatients at the three centres of India. All adults (≥15 years) with presumptive diagnosis of extra-pulmonary tuberculosis with no active pulmonary tuberculosis (TB), human immunodeficiency virus (HIV), or drug-resistant tuberculosis (DR-TB) were included in the study.

### 2.3. Data Collection

The basic sociodemographic information, start date of treatment, completion date of treatment, and type of EPTB case were recorded. All study participants were interviewed at the time of inclusion and closely followed until a diagnosis was reached, and additional visits to health care providers were documented at 2, 6, 9, and 12 months. Participants were included from various departments, such as genitourinary TB from the Obstetrics and Gynaecology Department; urogenital TB from the Urology Department; musculoskeletal TB from the Orthopaedics Department; surgical TB from other surgical departments, including General Surgery, ENT, and Ophthalmology Departments; lymph node TB (LNTB) and pleural TB from the Medicine and Pulmonary Department; central nervous system (CNS) TB from the Neurology Department; abdominal TB from the Gastroenterology Department; and pericardial TB from the Cardiology Department. A trained health-care staff member under the guidance of the principal investigator interviewed the treating physician of the EPTB patient in the corresponding department. Here, they looked for the various gaps and barriers occurring during the diagnosis. A questionnaire specially designed to identify the gaps and barriers based on NTEP diagnostic and treatment guidelines (Supplementary File S1) was used. Questions were formulated to determine whether all efforts were made to microbiologically confirm the diagnosis in presumptive EPTB patients under NTEP guidelines. It included questions to determine whether the regimen chosen, dose and duration of the antitubercular treatment (ATT), use of adjuncts, and dose and duration of adjuncts, including steroids, were acceptable under NTEP guidelines. TB treatment was initiated and supervised by the concerned departments of the hospitals in all cases. Under NTEP, the principle of treatment for tuberculosis was to administer daily weight-based fixed-dose combinations (FDCs) of first-line ATT. Patients receiving care in the private sector could also access these FDCs through NTEP upon request.

### 2.4. Operational Definitions

#### 2.4.1. EPTB Patients

Definitions of EPTB case refers to a case of TB involving different organs other than the lung parenchyma. Various case definitions in diagnosis of EPTB case are mentioned in Table 1.

#### 2.4.2. Delays

**Figure 1:**
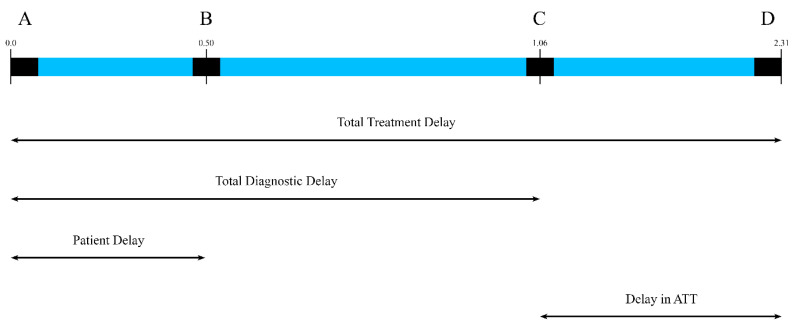
Illustration of different delays in EPTB diagnosis and treatment in months. A: onset of first symptom, B: time of first contact with HCP, C: confirmation of diagnosis, D: ATT started. Patient delay (A to B): Duration from onset of first symptom to first investigation. Health care system diagnostic delay (B to C): The time from the first contact to confirmatory diagnosis. Total treatment delay (A to D): Duration from first symptom to initiation of ATT.

Different types of delays observed in the diagnosis and treatment of EPTB case were assessed using patient delay, healthcare system diagnostic delay and total treatment delay. The definitions of these delays are provided in Table 2 and illustrated in Figure 1.

#### 2.4.3. Survival Outcome

A patient was classified as “dead” if it was documented in the hospital’s electronic records or reported during a follow-up phone call from any cause. Patients were considered “survived” if they either responded to phone follow-ups or had ongoing documentation in the hospital records through the study’s conclusion.

#### 2.4.4. Protocol for Follow-Up

We conducted a structured and comprehensive follow-up for patients diagnosed with EPTB in this study, ensuring regular assessment of clinical progress, response to therapy, and early identification of complications. Patients were scheduled for follow-up visits at predefined intervals—2 months, 6 months, 9 months, and 12 months—across all forms of EPTB. These visits were critical for tracking symptom resolution, weight gain, improvement in appetite, and adverse drug reactions (ADRs). Each follow-up visit included a detailed clinical evaluation, including assessment of ongoing symptoms and general health status. Laboratory investigations such as erythrocyte sedimentation rate (ESR) and C-reactive protein (CRP) were recorded if performed to monitor inflammatory markers. Additionally, radiological assessments were recorded based on the site of involvement; these included ultrasound (USG), computed tomography (CT), positron emission tomography-computed tomography (PET-CT), magnetic resonance imaging (MRI), and specialised investigations like echocardiography for pericardial TB and intravenous pyelography (IVP) for urogenital TB. For CNSTB cases, repeat cerebrospinal fluid (CSF) analysis was recorded if performed in certain cases to assess disease progression. In pleural TB, follow-up visits determined whether additional pleural taps were required. Surgical EPTB cases underwent imaging and clinical evaluations to assess the necessity for further intervention. Nutritional support was a critical part of the follow-up, with dietary recommendations provided to all patients, either formally or informally. Treatment adherence was reinforced, and the basis for stopping ATT was carefully determined using clinical assessment, imaging findings, or adherence to fixed-duration guidelines set by the national tuberculosis program. This structured follow-up ensured optimal treatment outcomes while minimising the risk of complications or treatment failure.

#### 2.4.5. Sample Size Calculation

The sample size for the study was calculated to estimate the prevalence of adherence to NTEP diagnostic and treatment guidelines in EPTB patients in surgical and non-surgical specialties separately, based on an expected prevalence of adherence of 70% based on a similar study by Chandru BA et al. [9]. To achieve an absolute precision of 5% and a level of significance of 5%, a minimum 323 EPTB patients was calculated. Therefore, a total of 433 EPTB patients were recruited from the three study sites, anticipating a 25% non-response rate.

#### 2.4.6. Statistical Analysis

For statistical analysis, Statistical Package for Social Sciences, version 28.0.1.0 (IBM Corp), was used. To analyse factors associated with mortality, odds ratios (ORs) for categorical variables were calculated using binary logistic regression. ORs were considered statistically significant at the 5% level if the 95% confidence intervals (CIs) did not include 1.0. Adjusted odds ratios (aORs) were calculated by including all the variables that were significant in a stepwise binary logistic regression model, and statistical significance was defined in the same way as for ORs. For analyses of mortality and diagnostic/treatment delays, the Mann–Whitney test was applied for group comparisons, as the data were non-normally distributed. A *p*-value < 0.05 was considered statistically significant.

#### 2.4.7. Ethical Consideration

The study was approved by the Institutional Ethics Committee of the All India Institute of Medical Sciences, Ansari Nagar, New Delhi with Ref No. IEC-153/04.02.2022, RP-29/2022. The study details were explained to the participants and written consent was obtained from them.

## 3. Results

**Figure 2:**
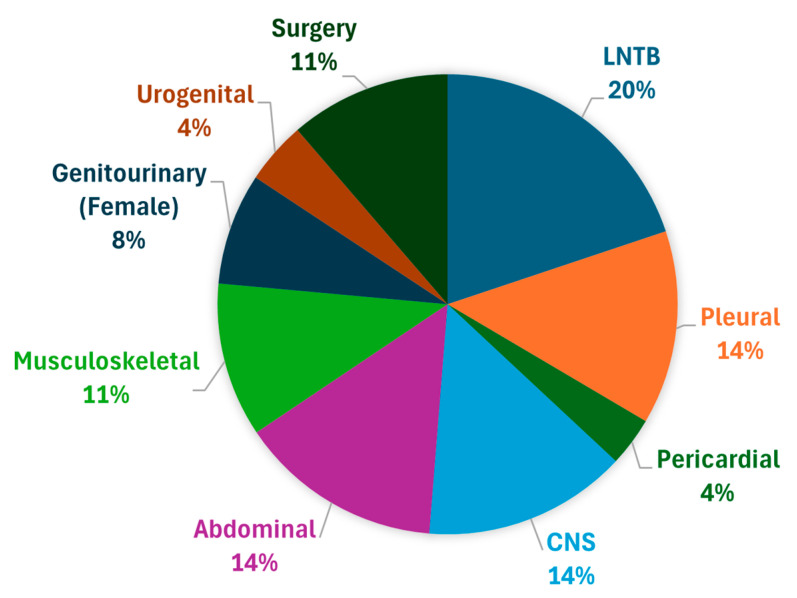
Distribution of EPTB in various systems.

Out of 487 patients screened, this study included a total of 433 patients diagnosed with EPTB. Among the different forms of EPTB, lymph node tuberculosis was the most common presentation (86 cases), followed by CNS tuberculosis (62 cases) and abdominal tuberculosis (62 cases). Other forms included musculoskeletal TB (47 cases), pleural TB (59 cases), genitourinary TB (34 female and 19 male cases), surgical TB (49 cases), pericardial TB (15 cases), and miscellaneous forms (Figure 2).

### 3.1. Patient Characteristics

The study comprised 205 males and 228 females, with a mean age of 34.1 years (range: 8–80 years), with a median age of 30 years. Sociodemographic details are shown in Table 3. Most patients lived in an urban area. Diabetes was reported in only 42 (10%) patients and history of tuberculosis was observed in 61 (15%) patients. Most of the patients enrolled had no chronic lung disease, with only 4% having history of other respiratory febrile illness in past 6 months.

### 3.2. Primary Outcome

**Figure 3:**
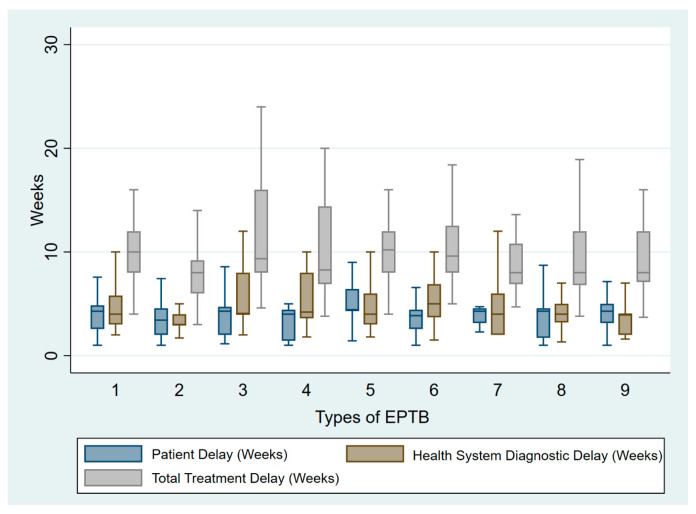
Box plot showing patient delay, health system diagnostic delay, and total treatment delay among various types of EPTB. Types of EPTB: 1—Abdominal TB, 2—CNS TB, 3—Genitourinary TB (female), 4—Urogenital TB, 5—LNTB, 6—Musculoskeletal TB, 7—Pericardial TB, 8—Pleural TB, 9—Surgical TB.

A total of 433 patients with EPTB were analysed. In 80% of patients enrolled, diagnosis of EPTB was suspected on the first day of contact. The median time from first symptom to presentation to the hospital, i.e., patient delay, was 4.2 weeks, while some patients presented after 21 weeks from the first symptom. The median health system diagnostic delay for all EPTB was 4 weeks. The maximum health system diagnostic delay was observed in surgical departments in making a diagnosis of EPTB. A maximum health system diagnostic delay of 10–14 weeks was observed in abdominal, surgical, genitourinary, urogenital, and musculoskeletal TB. Musculoskeletal TB and intestinal TB showed the lowest histopathological confirmation rate, suggesting significant barriers to obtaining tissue samples. In many cases of EPTB, it is often difficult to obtain an adequate sample for pathological and microbial testing. Thus, diagnosis often requires clinical judgements. Such delays can lead to disease progression, complications, and higher healthcare costs. The total treatment delay was 10.1 weeks, with a maximum delay of 28 weeks, in patients with musculoskeletal TB (Figure 3).

Although 282 patients (65%) were evaluated for microbiological evidence of tuberculosis by smear, culture, or Xpert/MTB-Rif, confirmatory diagnosis was made in 76% of cases clinically or radiologically without microbiological confirmation. NTEP guidelines emphasise histopathological diagnosis wherever possible, but it was observed that 125 patients (29%) underwent histopathology-based diagnosis, even though biopsy/FNAC (fine-needle aspiration cytology) is crucial for confirming EPTB in suspected cases. This suggests barriers in tissue sampling, processing, and reporting. A total of 38.7% of cases had imaging-based confirmation. In types of EPTB like LNTB, CNS TB, and pleural TB, pericardial TB had relatively high rates of microbiological testing, with FNAC, lumbar puncture, pleural fluid analysis, and pericardiocentesis performed in almost all patients, with a sample sent in a majority of cases for ADA and Xpert/MTB-RIF for confirmation of TB. LNTB, on the other hand, demonstrated better adherence to diagnostic guidelines, with 92% of cases undergoing microbiological testing; however, due to the indolent course of disease, lesser local symptoms, and lesser or no constitutional symptoms in LNTB, a longer patient delay, with a maximum of 21.5 weeks of delay from the first symptom to the first visit to the hospital, was observed.

Advanced diagnostic tests for EPTB, such as polymerase chain reaction (PCR) assays, Xpert/MTB-RIF, MGIT TB culture, and molecular methods were not readily available in many centres. Many patients who were referred from outside our institution with suspicion of EPTB were lacking a report of Xpert/MTB-RIF due to non-availability of testing at various centres where they were previously evaluated or the high cost of the test in the private sector and labs. In our study, Xpert/MTB-RIF was not sent in 18% of patients suspected to be EPTB-positive, with the highest rate of no molecular diagnosis seen in EPTB cases of surgical departments (36% in surgical TB and 28% in musculoskeletal TB). Relatively high rates of molecular testing with FNAC, lumbar puncture, pleural fluid analysis, and pericardiocentesis were found in LNTB (92%), CNS TB (80%), pleural TB (95%), and pericardial TB (87%), respectively. A total of 61.3% were diagnosed without imaging, raising concerns about reliance on empirical treatment. Diagnostic delays were also observed due to long waiting lists for radiological or invasive procedures such as ultrasound-guided or CT-guided biopsies, and invasive procedures such as colonoscopy guided biopsies in tertiary care centres. Most of these practitioners were aware of the Ni-Kshay portal method of TB notification and 383 patients (88%) were reported on the Nikshay portal. The gaps in diagnosis and treatment of different EPTB patients are shown in Table 4 and Table 5.

#### 3.2.1. CNS Tuberculosis

The mean duration of treatment was 11.4 ± 7.9 months. It was observed that meningitis was the most common subtype (53%), followed by tuberculoma and arachnoiditis. The majority had disseminated TB, while 26 cases (41.9%) were isolated to the CNS. Diagnostic evaluation included lumbar puncture in 56 cases (90%), ZN staining in 44 cases (70%), ADA in 47 cases (76%), Xpert MTB/Rif in 49 cases (73%), and MGIT TB culture in 47 cases (76%). Four patients (16.7%) did not undergo any microbiological confirmation prior to starting treatment. Imaging modalities were used in all cases for diagnosis, which included MRI (40%), CT scan (24%), CT + MRI (18%), and PET scan (1.6%), highlighting the role of imaging in CNS TB diagnosis and management. It was observed that only 42% of patients received steroids, which is a deviation from the national guidelines. Due to high drug intolerance or complications, therapy modification was required in 23 cases (37%).

#### 3.2.2. Abdominal Tuberculosis

This study included 62 cases of abdominal TB, with peritoneal TB (38 cases, 61%) being more common than intestinal TB (24 cases, 39%). Diagnostic methods included ascitic fluid sampling in 40 cases (64.5%), ZN staining in 29 cases (46.7%), and Xpert MTB/Rif in 43 cases (69.3%). USG of the abdomen was performed in 47 cases (75.8%), highlighting the role of radiology in diagnosis. After initiation of ATT, nine patients developed adverse effects, with vomiting the most common adverse reaction seen in eight patients and one patient developed jaundice. Therapy was modified in seven cases, indicating significant drug-related complications. The average treatment duration was 9 months, with treatment being stopped based on a clinical decision in 32 cases (51.6%).

#### 3.2.3. Lymph Node TB

LNTB was the most common type of EPTB observed. The most frequently involved site was cervical lymph nodes (62 cases, 72%), followed by axillary lymph nodes (11 cases, 12.7%). Most of the patients presented without pain and a gradually enlarging lymphadenopathy, which did not suppurate. A total of 70% of the patients presented after more than 4 weeks of noticing the swelling and 80% patients did not have any constitutional symptoms; thus, HCP considered it to mimic malignancy in some, or sarcoidosis or bacterial abscesses in others, leading to a delay in the suspicion of EPTB. Diagnostic evaluation included lymph node FNAC in 85 cases (99%), microbiological testing in 79 cases (92%), AFB testing in 62 cases (72%), and Xpert MTB/Rif in 72 cases (92%). Imaging modalities used for radiological diagnosis were chest X-ray (44%), ultrasound of a specific site (26%), and CECT scan (15%). LNTB demonstrated better adherence to diagnostic guidelines, but due to the indolent course of disease, lesser local symptoms, and lesser or no constitutional symptoms in LNTB, there was a longer patient delay, and a maximum of 21.5 weeks of delay from the first symptom to the first visit to the hospital was observed. Nine patients experienced adverse effects from ATT, and therapy was modified in six cases (7.5%). The average duration of treatment was 8.9 months, with treatment stopped based on clinical improvement in 35 cases (87.5%), radiological evidence in 4 cases (10.0%), and other reasons in 1 case (2.5%).

#### 3.2.4. Musculoskeletal TB

The study included 47 cases of musculoskeletal TB, with 41 cases (87.23%) isolated and 6 cases (12.7%) disseminated. Patients’ most common symptom was chronic back pain (95%) without constitutional symptoms. Neurological deficits appeared late, and paravertebral abscesses were not suspected on presentation and were usually diagnosed late on radiological investigations. Radiological imaging such as X-ray of the involved bone/joint was performed in all cases, with 27 patients (57.4%) also undergoing MRI for diagnosis. Monoarticular involvement was seen in 11 patients, especially of the knee or hip, with minimal pain or systemic signs that mimic rheumatoid arthritis, septic arthritis, or neoplasms. Invasive methods for histopathological diagnosis such as FNAC/biopsy of the site involved were performed in 30 cases (63.8%). In 29 (61.7%) cases, ATT was started before confirmatory microbiological diagnosis, 11 patients (23.4%) developed adverse effects, and therapy was modified in 5 cases (10.6%). The average treatment duration was 13.4 months, and treatment was stopped based on a clinical decision.

#### 3.2.5. Pericardial TB

Patients presented with slowly progressive pericardial effusion or constrictive pericarditis. The absence of overt TB symptoms in these patients led to misdiagnosis as viral pericarditis or malignancy. As per NTEP guidelines, pericardiocentesis was performed in all patients, with samples sent for AFB and ADA in all cases and Xpert/MTB-RIF performed in 11 cases (73%). All 15 patients (100.0%) underwent radiological imaging prior to treatment, emphasising the role of cardiac imaging. No adverse effects were observed, but therapy was modified in five cases (60%). The average treatment duration was 10 months. Corticosteroids along with ATT were given to 10 patients in accordance with national guidelines.

#### 3.2.6. Pleural TB

For diagnosis, pleural fluid analysis was performed in all cases, with samples sent for Xpert/MTB-RIF in 55 cases (93%) and for ADA in 55 cases (93%). The average treatment duration was 7 months, which was started before microbiological evidence in 17 cases (28%). Almost all cases (57 cases; 97%) had radiological imaging before starting treatment.

#### 3.2.7. Surgical TB

This study included 49 cases of surgical TB, with mostly painless swellings or masses that mimicked tumours and led to delayed presentation. Sinus formations were seen in patient with advanced disease and mostly had presented after prolonged antibiotics or multiple visits of being treated as osteomyelitis from different practitioners. FNAC of the affected site was performed in 30 cases (61%) and samples were sent for AFB (40%), Xpert/MTB-RIF (71%), and TB culture (42%). Six cases (31.6%) lacked microbiological confirmation before starting ATT. Radiological imaging was conducted in all cases with a USG or CECT scan of the affected site. ATT was started in 13 cases (26.5%) on a clinic-radiological basis. On ATT, four cases (0.08%) had adverse effects, with jaundice and skin effects, and therapy modification was required in five cases. The average treatment duration was 9 months, with a survival rate of 98%, while mortality occurred in 2%.

#### 3.2.8. Genitourinary TB (Female)

This study included 34 cases of female genitourinary TB and UPT was performed in all patients who visited OPD. In 89% of cases, it was entirely asymptomatic, followed by symptoms like chronic pelvic pain (62%) or infertility (45%). As per HCP, it is frequently confused with endometriosis or pelvic inflammatory disease, leading to extensive workup in making a diagnosis of EPTB. It was observed that a diagnosis was often made retrospectively, following infertility workup or laparoscopic biopsy. Endometrial aspirate was sent for analysis in 27 cases (79.4%), with Xpert/MTB-RIF performed in 28 cases (82.3%). All 34 patients (100%) had radiological imaging prior to treatment initiation. Imaging modalities used were USG, MRI, and CECT pelvis. Most patients were started on ATT on a clinic-radiological basis (14, 41%), while 11 patients had histopathological confirmation. three cases had adverse effects and therapy modifications were performed. The average treatment duration was 7 months, and all 34 patients (100%) survived.

#### 3.2.9. Urogenital TB

This study included 19 cases of urogenital TB, with 16 cases (84.2%) isolated and 2 cases (10.5%) disseminated. The renal function test and urine microscopy were performed in all cases. Microbiological diagnosis with urine for AFB was sent in 16 cases (84.2%) and Xpert/MTB-RIF was performed in 18 cases (94.7%), with 13 cases (68.4%) starting treatment with microbiological confirmation. All 7 cases had radiological imaging before ATT initiation, and USG KUB was performed in 16 (84.2%) cases and CECT in 10 cases (52.6%). The average treatment duration was 7.7 months. ATT-induced jaundice was seen in two cases, with modification of therapy in them.

### 3.3. Survival Outcomes

During follow-up, 18 patients died but no significant difference was found in mortality based on age or gender. Despite the gaps in diagnosis and treatment, the overall outcomes were favourable, with a survival rate of 95.8%. However, the highest number of mortalities, i.e., 11 cases, was noted in CNS TB.

Cases with comorbidities such as diabetes mellitus and history of tuberculosis had significantly higher odds of mortality as compared to those who did not have comorbidities, with an adjusted OR of 7.57 (95% CI: 2.64–21.72) and 4.82 (95% CI: 1.69–13.7), respectively.

As shown in Table 6, no significant difference was found in any delays (patient delay, health system diagnostic delay, or total treatment delay) between the surviving and deceased EPTB cases.

## 4. Discussion

This study highlights that many EPTB cases in India experienced delays in diagnosis and treatment from the onset of the symptoms. In our study, the patient delay and health system diagnostic delay both contributed almost equally to the total treatment delay. Many studies have observed similar findings of longer diagnostic and treatment delays in EPTB as compared to cases of pulmonary TB [10,11,12,13]. Many factors contribute to patient delays and delayed diagnosis, such as atypical presentation with fewer local symptoms, insidious onset of disease, and no constitutional symptoms, along with involvement of various sites of disease, lack of diagnostic facilities at peripheral hospitals, and the need for invasive interventions, such as lumbar puncture, pleural fluid analysis, FNAC, biopsy, and colonoscopy, which can cause health system delays [14,15,16].

### 4.1. Gaps in Diagnosis of EPTB Case

In our study, a median delay of 4 weeks was observed in confirmatory diagnosis, which was also observed in studies from India [17], Nepal [18], Ethiopia [19], and Africa [20]. There are no defined criteria for delays in diagnosis, which is unacceptable per NTEP guidelines. NTEP places emphasis on microbiological confirmation and histopathological confirmation wherever possible; in our study, the diagnosis of EPTB was suspected in 80% of the patients on day of contact, and 65% of cases had undergone microbiological investigation (smear, culture, Xpert/MTB-RIF) and 29% cases underwent histopathological investigation at presentation. Most of the cases (60%) were confirmed as EPTB on a clinic-radiological basis. For confirmatory diagnosis, heavy dependence on clinical-radiological diagnosis without microbiological support was observed, which is a deviation from NTEP guidelines [21] and raises concerns about the potential for misdiagnosis and overtreatment. The variation in diagnostic approaches across different types of EPTB is due to several reasons, and it has been observed that many patients were not diagnosed despite reaching health facilities. Some cases are known to have higher chances of missed diagnosis due to the lower sensitivity of many of the available diagnostic tests [22].

In types of EPTB like LNTB, CNS TB, and pleural TB, pericardial TB had relatively high rates of microbiological testing, with FNAC, lumbar puncture, pleural fluid analysis, and pericardiocentesis performed in almost all patients, with a sample sent in the majority of cases for ADA and Xpert/MTB-RIF for confirmation of TB. The diagnosis of EPTB frequently depends on patients completing complex, multi-step diagnostic processes, which are often associated with significant delay [23].

Musculoskeletal TB and intestinal TB showed the lowest histopathological confirmation rate, suggesting significant barriers to obtaining tissue samples. Lymph node TB, on the other hand, demonstrated better adherence to diagnostic guidelines, with 92% of cases undergoing microbiological testing [7].

This variability in adherence to microbiological diagnosis highlights site-specific challenges in sample collection and testing. The site of EPTB and the need for surgical or invasive sampling for its confirmatory diagnosis can cause health system delays; for example, as observed in our study, musculoskeletal, genitourinary, and LNTB had maximum health system diagnostic delays of 28, 24, and 24 weeks, respectively. In many high-TB-burden countries, advanced diagnostic tools such as Xpert MTB/RIF have not been widely implemented at the most decentralised healthcare level and vary considerably, both in terms of the criteria for their usage and in their distribution between urban and rural regions [24,25]. As a result, many individuals with TB are not receiving access to the most accurate diagnostic tests.

### 4.2. Gaps in Treatment of EPTB Patients

A high threshold in the initiation of treatment and awaiting diagnostic proof was observed as a factor for delays in management of EPTB in surgical TB (maximum: 24 weeks), urogenital TB (maximum: 26.5 weeks), musculoskeletal TB (maximum: 26.5), and genitourinary TB (maximum: 20 weeks), as seen in Table 4. The management of EPTB is further challenged by the practice of empirical treatment by healthcare providers. In our study, a significant number of patients were started on ATT without prior microbiological confirmation. This approach may lead to overtreatment, contributing to the development of antibiotic resistance, as well as increased morbidity and mortality.

Adverse effects of ATT were observed in 16% of cases, with jaundice being the most common, followed by skin rash, neuropathy, cytopenia, and vision loss. Similar findings have been observed in other studies, leading to modification of ATT during treatment [26]. In our study, CNS TB had the highest therapy modification rate, indicating high intolerance to first-line ATT drugs [27]. Due to symptoms such as blurring of vision, difficulty in assessment of vision in altered patients, and patients with optochiasmatic involvement, first-line ATT drugs such as Ethambutol were not prescribed to CNS TB patients. A survey conducted among neurologists in India revealed notable discrepancies between guideline-recommended treatment durations and those followed in clinical practice. Most neurologists reported a preference for extending therapy to 18 months or more [28]. Pericardial TB also required therapy modifications in 33% of cases, likely due to complications or intolerance. The average duration of ATT varied significantly, with bone TB requiring the longest treatment, with a mean duration of 13.4 ± 5.4 months, followed by CNS TB, with 11.4 ± 7.9 months [29].

### 4.3. Determinants of Survival Outcome

Despite the gaps in diagnosis and adherence to NTEP guidelines, the overall treatment success rate was high, with survival rates above 95.8% across most EPTB subtypes. Our study highlights the limited availability of published data from India regarding treatment success rates among patients with EPTB. We observed an overall treatment success rate of 95.8% in our cohort, which exceeds both the WHO’s target of at least 90% and the findings of an earlier Indian study that reported a success rate of 90.5% [30]. Additionally, our results surpass those of a study from Pakistan, which documented a treatment success rate of 71.1% among EPTB patients [4]. Several factors may contribute to lower treatment success rates, including poor adherence to anti-tuberculosis therapy, adverse drug reactions, limited patient awareness of the consequences of treatment interruption, and geographic barriers to accessing care [31]. Enhancing the quality of healthcare services, ensuring strong policy support, and fostering social and emotional support from the family, peers, and healthcare providers are crucial for improving treatment adherence and outcomes. Improved adherence may reduce treatment failure, relapse, disease transmission—particularly to vulnerable populations—and the emergence of drug resistance and mortality. Consistent with our findings, a study by Berihe Hiluf et al. identified that patients aged 35–44 years had a significantly higher risk of unsuccessful treatment outcomes, with a risk ratio of 1.61 [32]. Older individuals (above 35 years) tend to have comorbid conditions such as malnutrition, diabetes, and cancer, which may negatively impact their treatment response and lead to poorer outcomes [33]. A significant association between mortality and comorbidities such as DM and history of previous TB was observed in our study. Poor outcomes in these patients could be due to immune deficiency triggered by diabetes [34,35]. In our study, it was observed that there were increased deaths in CNS TB as compared to other EPTB types. Similar studies have reported unfavourable treatment outcomes in cases of CNS TB, highlighting the diagnostic challenges associated with this condition. These difficulties persist even when patient symptoms are recognised early, with additional risk factors—such as HIV co-infection and disseminated TB—further contributing to poor clinical outcomes [36]. Although considerable research has been conducted to explore the causes of poor treatment outcomes, there remains a need to focus more on underexplored risk factors—such as nutritional deficiencies, untreated depression, lack of social support, and insufficient awareness about EPTB—which may significantly impact treatment success and survival.

It is also important to examine the diagnostic pathways and healthcare provider practices to address delays in diagnosis, particularly within centralised laboratories equipped for EPTB testing.

## 5. Conclusions

Despite the challenges, the overall treatment success rate remains high, but adverse drug reactions and therapy modifications are frequent, necessitating closer monitoring and individualised management strategies. Patient delays as well as long health system delays in EPTB were observed leading to total treatment delays in EPTB. Various factors observed, such as atypical presentation, site of disease, lack of laboratory facilities, the need for surgical interventions, particularly in cases requiring invasive sampling (such as CNS TB, genitourinary, and musculoskeletal TB), high dependence on radiological investigations and equipment, and disease severity and complications, indicate barriers to timely diagnosis and treatment. To enhance EPTB diagnosis and management, there is a pressing need for improved access to microbiological testing, enhanced physician training on adherence to NTEP guidelines, and greater utilisation of imaging and histopathological techniques. Addressing these gaps will ensure timely diagnosis, appropriate treatment, and better treatment outcomes, contributing to tuberculosis elimination programmes.

## Figures and Tables

**Table 1:** Case definitions.

Term	Definition
Microbiologically confirmed TB	A presumptive TB patient with a biological specimen positive for AFB, or positive for MTB on culture, or positive for TB through Quality Assured Rapid Diagnostic molecular test.
Clinically diagnosed TB case	A presumptive TB patient who is not microbiologically confirmed but diagnosed with active TB by a clinician based on X-ray, histopathology, or clinical signs, with a decision to treat with a full course of anti-TB treatment.
Presumptive EPTB case	A patient with symptoms and signs of EPTB who needs to be investigated.
Bacteriologically confirmed case	A patient with a microbiological diagnosis of EPTB, based on positive microscopy, culture, or a validated PCR-based test. Note: A presumptive case started on ATT empirically, without microbiological testing, is considered a clinically diagnosed case. If subsequently found bacteriologically positive, the case is reclassified as bacteriologically confirmed.
Non-EPTB case	A patient was investigated for EPTB and diagnosed with another condition, with no microbiological evidence of EPTB.
Surgical EPTB	A patient with TB infections affecting the ear, nose, throat, and related head and neck structures, or having ulcers/discharging sinuses over lymph nodes, bones and joints, or ocular structures.

**Table 2:** Operational definitions of diagnostic and treatment delay.

Term	Definition
Patient delay	Duration from symptom onset to first visit with a health care provider (HCP), measured in weeks.
Healthcare system diagnostic delay	Time from first visit with a healthcare professional to confirmatory diagnosis, measured in weeks.
Total treatment delay	Time from onset of first symptom to initiation of ATT, measured in weeks.

**Table 3:** Sociodemographic characteristics of EPTP patients.

Demographic Parameters		(n = 433)	n (%)
Age	(Mean ± SD) Years	34.1 ± 14.24	
Gender	Male	205	47
	Female	228	53
Residence	Urban	319	74
	Rural	114	26
History of TB	No	333	85
	Yes	61	15
History of current chronic lung disease	No	426	98
	Yes	7	2
History of Diabetes Mellitus	No	391	90
	Yes	42	10

**Table 4:** Gaps in diagnosis of different EPTB subpopulations.

EPTB Type (n = 433)	Patient Delay Median (min, max) (Weeks)	Health Care system Diagnostic Delay Median (min, max) (Weeks)	Total Treatment Delay Mean **±** SD (Weeks)	No Microbiological Diagnosis, n (%)	No Radiological Investigations n (%)	No HIV Testing n (%)	Payment Was Needed n (%)
Lymph node TB (n = 86)	4.4 (1.4–21.5)	4 (1.8–10)	11.1 ± 4.4	7 (8)	9 (10)	8 (9)	9 (10)
CNS TB (n = 62)	3.4 (1–17.4)	3 (1.2–10.3)	8.2 ± 3.4	27 (4)	0	3 (5)	7 (11)
Abdominal TB (n = 62)	4.2 (1–21.8)	4 (2–14)	10 (4–20)	15 (24)	0	5 (8)	3 (5)
Pleural TB (n = 59)	4.2 (1–21)	4 (1.3–9.8)	8 (3.8–26.5)	9 (15)	2 (3)	3 (5)	7 (12)
Surgical TB (n = 49)	4.2 (1–17.4)	4 (1.6–12)	8 (3.7–20)	14 (28)	0	7 (14)	7 (14)
Musculoskeletal TB (n = 47)	3.8 (1–16.5)	5 (1.5–10)	9.6 (5–28)	28 (60)	1 (0.02)	5 (11)	5 (11)
Genitourinary TB Female (n = 34)	4.2 (1.1—8.5)	4 (2–12)	9.35 (4.6–24)	7 (20)	13 (38)	4 (12)	1 (3)
Urogenital TB (n = 19)	4 (1–14.4)	4.2 (1.8–10)	8.2 (3.8–20)	5 (26.3)	2 (10)	2 (10)	1 (5)
Pericardial TB (n = 15)	4.2 (1–21)	4 (1.3–9.8)	8 (3.8–26.5)	3 (20)	0	0	4 (27)
*p*-value	0.0014	0.013	0.0005	<0.001	<0.001		

*p*-value ≤ 0.005 is considered significant.

**Table 5:** Gaps in treatment of different subpopulations of EPTB.

EPTB Type (n = 433)	1st Line ATT Not Initiated n (%)	Mean Duration of Treatment Mean **±** SD (Months)	No Pyridoxine Supplementation n (%)	Modified ATT n (%)	Adverse Effects n (%)
CNS TB (n = 62)	18 (29)	11.4 ± 7.9	0	23 (37)	10 (16)
Abdominal TB (n = 62)	21 (33)	9 ± 4.50	0	7 (11)	9 (15)
Lymph node TB (n = 86)	3 (3)	7.9 ± 3.07	9 (10)	6 (7)	9 (10)
Musculoskeletal TB (n = 47)	12 (25)	13.4 ± 5.40	1 (2)	5 (11)	11 (23)
Pericardial TB (n = 15)	0	9.5 ± 5.30	0	5 (33)	1 (0.06)
Pleural TB (n = 59)	31 (52)	7.1 ± 2.26	14 (23)	5 (8)	21 (35)
Surgical TB (n = 49)	11 (22)	9.6 ± (4.3)	0	5 (10)	4 (8)
Genitourinary TB Female (n = 34)	0	7.11 ± 1.64	1 (3)	3 (9)	3 (9)
Urogenital TB (n = 19)	1 (15)	7.78 ± 3.5	0	2 (10)	2 (10)

**Table 6:** Parameters of EPTB patients according to survival outcomes.

Characteristics	Values	Died (N = 18)	Survived (N = 415) n (n%)	Unadjusted R (95%CI)	*p*-Value	Adjusted OR (95%CI)	*p*-Value
Age (Years)	<34	8 (44)	250 (60)	1			
	>34	10 (56)	165 (40)	1.89 (0.73, 4.90)	0.188		
Gender	Male	10 (56)	186 (45)				
	Female	8 (44)	229 (55)	0.65 (0.25, 1.68)	0.374		
History of DM	Yes	7 (39)	38 (9)		<0.001	7.57 (2.64–21.72)	<0.001
	No	11 (61)	377 (91)	6.31 (2.31, 17.24)			
History of TB disease	Yes	7 (39)	58 (14)		0.007	4.82 (1.69–13.7)	<0.003
	No	11 (61)	357 (86)	3.91 (1.46, 10.51)			
Patient delay (weeks)	<4.43	12 (67)	288 (69)	1			
	≥4.43	6 (33)	127 (31)	1.13 (0.42, 3.09)	0.806		
Health system delay (weeks)	<4.53	10 (56)	275 (66)				
	≥4.53	8 (44)	140 (34)	1.57 (0.60, 4.07)	0.352		
Total treatment delay (weeks)	<10.1	10 (56)	254(61)	1			
	≥10.1	8 (44)	161 (39)	1.26 (0.48, 3.26)	0.631		

## Data Availability

All relevant data can be found in the manuscript and the Supplementary Materials Files.

## Supplementary Materials

The following supporting information can be downloaded at: https://www.mdpi.com/article/10.3390/tropicalmed10080206/s1.
