# Supplementary material for: Evaluating the Gaps in the Diagnosis and Treatment in Extra-Pulmonary Tuberculosis Patients Under National Tuberculosis Elimination Programme (NTEP) Guidelines: A Multicentric Cohort Study
Source: Trop Med Infect Dis. 2025 Jul 24;10(8):206. doi: 10.3390/tropicalmed10080206 (PMC12390147; doi:10.3390/tropicalmed10080206)
Supplement: Supplementary file 1 [file tropicalmed-10-00206-s001.zip › tropicalmed-3667786-supplementary.pdf]

Screening Proforma

CENTRAL CASE NUMBER:…………………	UHID NUMBER:…………………

Study title: "To evaluate the gaps in the diagnosis and treatment in extra-pulmonary tuberculosis patients under National Tuberculosis Elimination Programme (NTEP) guidelines among various multi-specialties: a multicentric observational study."

Table S1: THE FOLLOWING DATA TO BE COLLECTED AFTER CONSENT IS OBTAINED:

**A.**	**PATIENT INFORMATION**		
1.	Name of patient:	Age:	Date of signed Informed Consent Form
2.	Father's / guardian's name	Gender: M/F	Date of interview
3.	Phone No:	Email:	Name of interviewer
**B.**	**SOCIO-DEMOGRAPHIC PROFILE**		
1.	House No.:	District:	State:
	Street/Colony:	Village/Mohalla:	Pin code:
2.	Religion:	Nationality:	
**C.**	**Baseline procedures and data collection**		
	**Baseline data obtained**	**Response**	**If response yes kindly specify***
	History of TB disease	Yes/No	
	History of BCG Vaccination & Number of BCG scars (by visual/physical examination)	Yes/No	
	Access to smartphone	Yes/No	

	History of previous HIV testing	Yes/No	
	Urine Pregnancy test result (if applicable)	Yes/No	
	Plans of pregnancy in 30 days	Yes/No	
	Current diabetes mellitus	Yes/No	
	Current chronic lung disease (Chronic obstructive lung disease or asthma or non-cystic fibrosis bronchiectasis)	Yes/No	
	Chemotherapy in past 3 months	Yes/No	
	Sputum for AFB	Yes/No	
	Sputum for Genexpert	Yes/No	
	Chest Xray	Yes/No	

*Mention the current state/class/disorder/treatment of the disease

History of other Respiratory febrile Illiness in the past six months?

Yes No

If Yes, Record the follow details

1. Frequency of Occurrence……………………………………………….
2. Duration of illness: ……………………………………………..
3. Hospitalized due to respiratory illness Yes No

If Yes,

Description:…………………………………………………………………………………………………

Implementation of NTEP & INDEX-TB guidelines: Constraints / Gaps

Title- **"To evaluate the gaps in the diagnosis and treatment in extra-pulmonary tuberculosis patients under National Tuberculosis Elimination Programme (NTEP) guidelines among various multi-specialties: a multicentric observational study."**

DETECTION OF EPTB

- ☐ Date of first contact with symptoms
- ☐ Was diagnosis of EPTB suspected on the Day of contact
- ☐ Which investigations were advised?
- ☐ Date of investigations advised
- ☐ Time from the first contact to confirmatory diagnosis

Which of the following Methods used for TB Notification

- ☐ Ni-kshay Portal
- ☐ Call Centre - Nikshay sampark
- ☐ Physical Registration form
- ☐ District Nodal Officers

NI-KSHAY ID

Which of the following Patient Support System were offered?

- ☐ Ni-Kshay Poshan Yojana (NPY)
- ☐ Transport support for TB patients in notified tribal areas
- ☐ Honorarium for Treatment Supporters
- ☐ Notification & Treatment Outcome Incentive for Private Sector Providers
- ☐ Pradhan Mantri TB Mukt Bharat Abhiyaan —Ni-K shay Mitra

Tools for Microbiological Confirmation —

1. Molecular (Genotypic) Testing: Nucleic acid amplification tests (NAATS) by Polymerase Chain
   - ☐ GeneXpert
   - ☐ TrueNat
   - ☐ Line Probe Assay (LPA)
2. Direct Microscopy:
   - ☐ Ziehl-Neelsen (ZN) staining
   - ☐ Fluorescent staining
3. Culture Lowenstein Jensen (LJ) medium or Automated liquid culture system like MGIT 960
4. Other test modalities to detect EPTB which is used by clinicians are:
   - ☐ Histopathology examination (Tissue biopsy)
     Biochemical examination of body fluids (for Adenosine Deaminase (ADA)
   - ☐ Radiological examinations like CXR, CT scan, USG, MRI and PET scan
5. Drug Susceptibility Test:
   - ☐ Phenotypic Drug susceptibility test (DST): BACTEC MGIT 960 – an Automated Liquid Culture System.
   - ☐ Molecular (Genotypic) DST

PROFORMA FOR ABDOMINAL TB

Title of the Research Project: To evaluate the gaps in the diagnosis and treatment in extra-pulmonary tuberculosis patients under National Tuberculosis Elimination Programme (NTEP) guidelines among various multi-specialties: a multicentric observational study.

DIAGNOSIS

- **Diagnosis: Peritoneal TB/Intestinal TB/Oesophageal TB/Gastroduodenal TB/Hepatobiliary TB/Pancreatic TB/Peri-anal TB**
- **Extent: Isolated abdominal TB/Disseminated TB**

Diagnostic modalities:

- Was ascitic fluid sampling done? Yes/No. If no, why?_________________________________
- Was ascitic fluid sent for cell count and differential? Yes/No. If no, why?________________
- Was ascitic fluid sent for protein/albumin? Yes/No. If no, why?_________________________
- Was ascitic fluid sent for AFB? Yes/No. If no, why?____________________________
- Did the patient have to pay for it? Yes/No. If yes, how much?_________________________
- Was ascitic fluid sent for malignant cytology? Yes/No. If no, why?_______________
- Was ascitic fluid sent for GeneXpert? Yes/No. If no, why?_____________________________
- Did the patient have to pay for it? Yes/No. If yes, how much?__________________________
- Was ascitic fluid for TB culture? Yes/No. If no, why?__________________________
- Was ascitic fluid sent for ADA? Yes/No. If no, why?___________________________
- Did the patient have to pay for it? Yes/No. If yes, how much?__________________________
- Was HIV testing done? Yes/No. If no, why?_________________________________
- Was chest X-ray done? Yes/No. If no, why?_________________________________
- Was USG abdomen done? Yes/No. If no, why?_________________________________
- Was CT/MRI of abdomen done? Yes/No. Why?_________________________________
- Was FNAC/biopsy done where feasible for microbiological diagnosis? Yes/No. If no, why?_________________________________
- In inconclusive cases, was laparoscopy and laparoscopy guided biopsy done for microbiological diagnosis? Yes/No. If no, why?_________________________________

For patients with suspected intestinal TB:

- Was the patient with suspected intestinal TB taken for ileocolonoscopy? Yes/No. If no, why?____________________
- Was biopsy taken from suspicious sites during ileocoloscopy? Yes/No. If no, why?_______________________________
- Was CT/MR enterography/enteroclysis done in patients with suspected intestinal TB? Yes/No. Why?________________________________
- Was UGI endoscopy done in patients with suspected intestinal TB? Yes/No. Why?________________________________
- Was barium studies done in patients with suspected intestinal TB? Yes/No. Why?________________________________

For all abdominal TB patients:

- On what basis was the anti-tubercular drugs started? (Tick the appropriate option)
    - o TB culture
    - o GeneXpert
    - o Histopathology (caseating granulomas)
    - o Acid fast bacilli on microscopy
    - o Adenosine deaminase
    - o Microbiological evidence of TB elsewhere
    - o Empirical ATT trial

TREATMENT

Date of starting TB Rx:_________________ Date of completing TB Rx:________________

- Was HRZE used in intensive phase? Yes/No. If no, why?_________________________
- Was any other agent added to the intensive phase apart from HRZE? Yes/No. If yes, why?________________________
- Was pyridoxine supplementation started? Yes/No. If no, why?________________________
- Was duration of continuation phase at least 4 months? Yes/No. If no, why?________________________
- Was the regimen HRE during continuation phase? Yes/No. If no, why?________________________
- Was the total duration of ATT at least 6 months? Yes/No. If no, why?________________________
- Time from first symptom to starting of the ATT _________________________________
- Was the patient started on steroids? Yes/No. If yes,
    a) What was the dose and duration of steroids used?_______________
    b) Reason for starting steroids?____________________________________

PROFORMA FOR BONE/JOINT TB

Title of the Research Project: To evaluate the gaps in the diagnosis and treatment in extra-pulmonary tuberculosis patients under National Tuberculosis Elimination Programme (NTEP) guidelines among various multi-specialties: a multicentric observational study.

DIAGNOSIS

- **Diagnosis: Spinal TB/Tubercular arthritis/TB osteomyelitis of any other bone**
- **Extent: Isolated musculoskeletal TB/Disseminated TB**

Diagnostic modalities:

- Was X-ray spine done? Yes/No. If no, why?________________________________
- Was MRI spine done in patients with suspected spinal TB? Yes/No. If no, why?________________________________
- Did the patient have to pay for it? Yes/No. If yes, how much?_________________________
- Was CT spine done in patients with suspected spinal TB? Yes/No. Why?________________________________
- Did the patient have to pay for it? Yes/No. If yes, how much?_________________________
- Was biopsy/FNAC of the lesion done? Yes/No. If no, why?________________________________
- Was the biopsy/FNAC done under imaging guidance? Yes/No. If no, why?________________________
- What was the imaging modality used to guide the FNAC?______________________
- Was the sample sent for gram stain? Yes/No. If no, why?_______________________
- Was the sample sent for histopathology? Yes/No. If no, why?________________________
- Was the sample sent for AFB? Yes/No. If no, why?___________________________
- Did the patient have to pay for it? Yes/No. If yes, how much?___________________
- Was the sample sent for GeneXpert? Yes/No. If no, why?______________________________
- Did the patient have to pay for it? Yes/No. If yes, how much?___________________
- Was the sample sent for TB culture? Yes/No. If no, why?______________________
- Was the sample sent for fungal culture? Yes/No. If no, why?___________________________
- Did the patient have to pay for it? Yes/No. If yes, how much?_________________________
- Was ATT started before sampling? Yes/No. If yes, why?__________________________
- Was HIV testing done? Yes/No. If no, why?________________________________
- Was chest X-ray done? Yes/No. If no, why?________________________________
- On what basis was the anti-tubercular drugs started? (Tick the appropriate option)
    - TB culture

- GeneXpert
- Histopathology (caseating granulomas)
- Acid fast bacilli on microscopy
- Microbiological evidence of TB elsewhere
- Empirical ATT trial based on clinic-radiological evidence

TREATMENT

Date of starting TB Rx:_______________ Date of completing TB Rx:____________

- Was HRZE used in intensive phase? Yes/No. If no, why?_________________________________
- Was any other agent added to the intensive phase apart from HRZE? Yes/No. If yes, why?_________________________________
- Was pyridoxine supplementation started? Yes/No. If no, why?_________________________________
- Was duration of continuation phase at least 10 months? Yes/No. If no, why?_________________________
- Was the regimen HRE during continuation phase? Yes/No. If no, why?_____________________________
- Was the total duration of ATT more than 12 months? Yes/No. If no, why?____________________________
- What was the total duration of treatment?___________________________
- Time from hospitalization to starting of the ATT _____________________
- Was weekly neural charting done to detect neural recovery or deterioration? Yes/No. If no, why?_____________________________
- Was repeat X-rays done every 3 months to detect recovery or deterioration? Yes/No. If no, why?_____________________________
- Were repeat MRI scans done at 6, 9, 12 and 18 months following initiation of treatment to assess healing? Yes/No. If no, why?____________________________
- Was the patient started on steroids? Yes/No. If yes,
  a) What was the dose and duration of steroids used?_______________
  b) Reason for starting steroids?_____________________________________
  - Any challenges faced in the treatment? Yes/No. If yes, specify?___________________

PROFORMA FOR CNS TB

Title of the Research Project: To evaluate the gaps in the diagnosis and treatment in extra-pulmonary tuberculosis patients under National Tuberculosis Elimination Programme (NTEP) guidelines among various multi-specialties: a multicentric observational study.

DIAGNOSIS

- **Diagnosis: TB meningitis/Cerebral and spinal tuberculoma/Myelitis/Arachnoiditis**
- **Extent: Isolated CNS TB/Disseminated TB**

Diagnostic modalities:

- Was lumbar puncture done? Yes/No. If no, why?________________________________
- Was CSF sent for cell count and differential within 30 minutes? Yes/No. If no, why?______________________
- Was CSF for cell count and differential processed? Yes/No. If no, why?______________________
- Was CSF sent for protein/glucose? Yes/No. If no, why?________________________
- Was simultaneous blood glucose level measured? Yes/No. If no, why?________________
- Was CSF sent for gram stain? Yes/No. If no, why?______________________________
- Was CSF sent for AFB? Yes/No. If no, why?________________________________
- Did the patient have to pay for it? Yes/No. If yes, how much?_________________________
- Was CSF sent for GeneXpert? Yes/No. If no, why?_____________________________
- Did the patient have to pay for it? Yes/No. If yes, how much?_____________________
- Was CSF sent for TB culture? Yes/No. If no, why?_____________________________
- Was CSF sent for ADA? Yes/No. If yes, why?_________________________________
- Did the patient have to pay for it? Yes/No. If yes, how much?_____________________
- Was HIV testing done? Yes/No. If no, why?________________________________
- Was chest X-ray done? Yes/No. If no, why?________________________________
- Was CECT brain done? Yes/No. If no, why?________________________________
- Was CEMRI brain done? Yes/No. If no, why?________________________________

- On what basis was the anti-tubercular drugs started? (Tick the appropriate option)
  - TB culture
  - GeneXpert
  - Histopathology (caseating granulomas)
  - Acid fast bacilli on microscopy
  - Adenosine deaminase
  - Microbiological evidence of TB elsewhere
  - Empirical ATT trial base on clinico-radiological evidence

TREATMENT

Date of starting TB Rx:_______________ Date of completing TB Rx:____________

- Time from first symptom to starting of the ATT___________________
- Was HRZE used in intensive phase? Yes/No. If no, why?________________________________

- Was any other agent added to the intensive phase apart from HRZE? Yes/No. If yes, why?_____
- Was pyridoxine supplementation started? Yes/No. If no, why?_____________________________
- Was duration of continuation phase at least 7 months? Yes/No. If no, why?__________________
- Was the regimen HRE during continuation phase? Yes/No. If no, why?_____________________
- Was the total duration of ATT at least 9 months? Yes/No. If no, why?____
- Was the patient started on steroids? Yes/No. If yes,
  a) What was the dose and duration of steroids used?________________
  b) Reason for starting steroids?_____________________________________

PROFORMA FOR FEMALE GENITAL TB

Title of the Research Project: To evaluate the gaps in the diagnosis and treatment in extra-pulmonary tuberculosis patients under National Tuberculosis Elimination Programme (NTEP) guidelines among various multi-specialties: a multicentric observational study.

DIAGNOSIS

- **Diagnosis: Female genital tract TB/Disseminated TB**

Diagnostic modalities:

- Was urine pregnancy test done? Yes/No. If no, why?________________________________
- Was pelvic ultrasound done? Yes/No. If no, why?___________________________
- Was hysterosalpingogram done? Yes/No. Why?______________________________
- Was CT pelvis done? Yes/No. Why?_________________________________
- Was MRI pelvis done? Yes/No. Why?_________________________________
- Was FDG PET-CT done? Yes/No. Why?_________________________________
- Was endometrial aspirate done? Yes/No. If no, why?__________________________
- Was the sample sufficient for analysis? Yes/No
- Was the sample sent for histopathology? Yes /No. If no, why?_________________
- Was the sample sent for AFB? Yes /No. If no, why?__________________________
- Was the sample sent for GeneXpert? Yes/No. If no, why?______________________
- Did the patient have to pay for the test? Yes/No. If yes, how much?_____________________
- Was the sample sent for TB culture? Yes /No. If no, why?_________________
- Was dilatation and curettage done in suspected endometrial TB? Yes/No. If no, why?__________________________
- Was the tissue sufficient for analysis? Yes/No
- In inconclusive cases, was hysteroscopy guided biopsy attempted? Yes/No. If no, why?_______________________
- Was the tissue sufficient for analysis? Yes/No
- In inconclusive cases, was laparoscopy guided biopsy attempted? Yes/No. If no, why?_______________________
- Was the tissue sufficient for analysis? Yes/No
- Was chest x-ray done? Yes/No. If no, why?_______________________________
- Was HIV testing done? Yes/No. If no, why?_______________________________
- On what basis was the anti-tubercular drugs started? (Tick the appropriate option)
    - o TB culture
    - o GeneXpert
    - o Histopathology (caseating granulomas)
    - o Acid fast bacilli on microscopy
    - o Microbiological evidence of TB elsewhere

- Laparoscopic findings of FGTB
- Hysteroscopic findings of FGTB
- PET-CT findings of FGTB
- Empirical ATT trial based on clinic-radiological evidence

TREATMENT

Date of starting TB Rx:________________ Date of completing TB Rx:_____________

- Was HRZE used in intensive phase? Yes/No. If no, why?_________________________________
- Was any other agent added to the intensive phase apart from HRZE? Yes/No. If yes, why?_________________________________
- Was pyridoxine supplementation started? Yes/No. If no, why?_________________________________
- Was the regimen HRE during continuation phase? Yes/No. If no, why?____________________________
- Was the total duration of ATT more than 6 months? Yes/No. If no, why?___________________________
- Time from onset of first symptom to starting of the ATT ______________________
- Was the patient started on steroids? Yes/No. If yes,
  a) What was the dose and duration of steroids used?________________
  b) Reason for starting steroids?_____________________________________

PROFORMA FOR LYMPH NODE TB

Title of the Research Project: To evaluate the gaps in the diagnosis and treatment in extra-pulmonary tuberculosis patients under National Tuberculosis Elimination Programme (NTEP) guidelines among various multi-specialties: a multicentric observational study.

DIAGNOSIS

- **Diagnosis: Isolated lymph node TB/Disseminated TB**
- **Site: Cervical/Axillary/Mediastinal/Abdominal/Inguinal**

Diagnostic modalities:

- Was lymph node FNAC done? Yes/No. If no, why?___________________________
- Was FNAC done under imaging guidance? Yes/No. If no, why?_____________________
- Was the sample sent for cytology? Yes/No. If no, why?________________________
- Was the sample sent for AFB? Yes/No. If no, why?___________________________
- Was the sample sent for GeneXpert? Yes/No. If no, why?____________________________
- Did the patient have to pay for it? Yes/No. If yes, how much?_________________________
- Was the sample sent for TB culture? Yes/No. If no, why?_______________________
- Was HIV testing done? Yes/No. If no, why?________________________________
- Was chest X-ray done? Yes/No. If no, why?________________________________
- Was USG or CT of the chest and abdomen done? Yes/No. Why?____________________________
- If FNAC was inconclusive, was excisional lymph node biopsy done? Yes/No. If no, why?_________________
- Was the FNAC/excisional lymph node biopsy done before commencing ATT? Yes/No. If no, why?_________________
- What was the imaging modality used to guide the FNAC? (Tick the appropriate response)
    - EBUS
    - EUS
    - CT-guided
    - USG-guided
    - Mediastinoscopy guided
    - Others, specify__________________
- On what basis was the anti-tubercular drugs started? (Tick the appropriate option)
    - TB culture
    - GeneXpert
    - Histopathology (caseating granulomas)
    - Acid fast bacilli on microscopy
    - Microbiological evidence of TB elsewhere
    - Empirical ATT trial based on clinic-radiological evidence

TREATMENT

Date of starting TB Rx:_______________ Date of completing TB Rx:____________

- Was HRZE used in intensive phase? Yes/No. If no, why?______________________________
- Was any other agent added to the intensive phase apart from HRZE? Yes/No. If yes, why?_________________________________
- Was pyridoxine supplementation started? Yes/No. If no, why?_________________________________
- Was duration of continuation phase at least 4 months? Yes/No. If no, why?__________________________
- Was the regimen HRE during continuation phase? Yes/No. If no, why?_____________________________
- Was the total duration of ATT more than 6 months? Yes/No. If yes, why?___________________________
- Was the total duration of ATT less than 6 months? Yes/No. If yes, why?___________________________
- Time from first symptom to starting of the ATT _____________________
- Was the patient started on steroids? Yes/No. If yes,
    a) What was the dose and duration of steroids used?_______________
    b) Reason for starting steroids?_____________________________________

PROFORMA FOR PERICARDIAL TB

Title of the Research Project: To evaluate the gaps in the diagnosis and treatment in extra-pulmonary tuberculosis patients under National Tuberculosis Elimination Programme (NTEP) guidelines among various multi-specialties: a multicentric observational study.

DIAGNOSIS

- **Diagnosis: Pericardial TB/Disseminated TB**

Diagnostic modalities:

- Was chest X-ray done? Yes/No. If no, why?________________________________
- Was HIV testing done? Yes/No. If no, why?________________________________
- Was ECG done? Yes/No. If no, why?_______________________________________
- Was CT chest done? Yes/No. Why?____
- Was it contrast enhanced CT? Yes/No. If no, why?__________________________
- Was there a need to get cardiac MRI? Yes/No. If yes, why?_________________________
- Was echocardiography (transthoracic) done? Yes/No. If no, why?______________________
- Was diagnostic pericardiocentesis done? Yes/No. If no, why?____________________
- Was the pericardial fluid sent for AFB? Yes/ No. If no, then why?__________________________
- Did the patient have to pay for it? Yes/No. If yes, how much?_____ ____________________
- Was the pericardial fluid sent for GeneXpert? Yes/ No. If no, then why?__________________________
- Did the patient have to pay for it? Yes/No. If yes, how much?_____ ____________________
- Was pleural fluid sent for TB culture? Yes/No. If no, why?_______________________
- Did the patient have to pay for it? Yes/No. If yes, how much?_________________________
- Was the pericardial fluid sent for ADA levels? Yes/ No. If no, then why?__________________________
- Did the patient have to pay for it? Yes/No. If yes, how much?_____ ____________________
- Was ATT started empirically before pericardiocentesis? Yes/NO. If yes, why?_____________________________
- On what basis was the anti-tubercular drugs started? (Tick the appropriate option)
    - TB culture
    - GeneXpert
    - Histopathology (caseating granulomas)
    - Acid fast bacilli on microscopy

- Adenosine deaminase
- Microbiological evidence of TB elsewhere
- Empirical ATT trial

TREATMENT

Date of starting TB Rx:________________ Date of completing TB Rx:____________

- Was HRZE used in intensive phase? Yes/No. If no, why?___________________________________
- Was any other agent added to the intensive phase apart from HRZE? Yes/No. If yes, why?___________________________________
- Were corticosteroids used along with ATT? Yes/ No. If no, why?____________________
- Was pyridoxine supplementation started? Yes/No. If no, why?___________________________________
- Was the regimen HRE during continuation phase? Yes/No. If no, why?______________________________
- Was the total duration of ATT more than 6 months? Yes/No. If no, why?_____________________________
- Time from onset of first symptom to starting of the ATT ______________________
- Was the patient started on steroids? Yes/No. If yes,
    a) What was the dose and duration of steroids used?________________
    b) Reason for starting steroids?_______________________________________

PROFORMA FOR PLEURAL TB

Title of the Research Project: To evaluate the gaps in the diagnosis and treatment in extra-pulmonary tuberculosis patients under National Tuberculosis Elimination Programme (NTEP) guidelines among various multi-specialties: a multicentric observational study.

DIAGNOSIS

- **Diagnosis: Pleural TB/Disseminated TB**

Diagnostic modalities:

- Was pleural tap done? Yes/No. If no, why?________________________________
- Was pleural tap under the imaging guidance? Yes/No. If no, why?_______________________
- What modality was selected for guiding pleural tap?__________________________
- Was pleural fluid sent for cell count and differential count? Yes/No. If no, why?_______________________
- Was pleural fluid sent for protein/glucose? Yes/No. If no, why?__________________________________
- Was pleural fluid sent for LDH? Yes/No. If no, why?_________________________
- Was serum simultaneously sent for LDH? Yes/No. If no, why?____________________________
- Was pleural fluid sent for malignant cytology? Yes/No. If no, why?_____________________
- Was pleural fluid sent for AFB? Yes/No. If no, why?__________________________
- Was pleural fluid sent for GeneXpert? Yes/No. If no, why?_____________________
- Did the patient have to pay for it? Yes/No. If yes, how much?_________________________
- Was pleural fluid sent for TB culture? Yes/No. If no, why?______________________
- Was pleural fluid sent for ADA level? Yes/No. If no, why?_____________________
- Did the patient have to pay for it? Yes/No. If yes, how much?_________________________
- Was CT chest and abdomen done? Yes/No. Why?____________________________
- Was USG chest done? Yes/No. Why?________________________________
- Was sputum work-up for TB done? Yes/No. Why?___________________________
- In inconclusive cases, was pleural biopsy done? Yes/No. If no, why?_______________________
- Was HIV testing done? Yes/No. If no, why?_______________________________
- Was chest X-ray done? Yes/No. If no, why?_______________________________
- On what basis was the anti-tubercular drugs started? (Tick the appropriate option)
    - TB culture
    - GeneXpert
    - Histopathology (caseating granulomas)
    - Acid fast bacilli on microscopy

- Adenosine deaminase
- Microbiological evidence of TB elsewhere
- Empirical ATT trial based on clinico-radiological basis

TREATMENT

Date of starting TB Rx:________________ Date of completing TB Rx:_____________

- Was HRZE used in intensive phase? Yes/No. If no, why?___________________________________
- Was any other agent added to the intensive phase apart from HRZE? Yes/No. If yes, why?___________________________________
- Was pyridoxine supplementation started? Yes/No. If no, why?___________________________________
- Was duration of continuation phase at least 4 months? Yes/No. If no, why?__________________________
- Was the regimen HRE during continuation phase? Yes/No. If no, why?______________________________
- Was the total duration of ATT at least 6 months? Yes/No. If no, why?_____________________________
- Time from first symptom to starting of the ATT ______________________
- Was the patient started on steroids? Yes/No. If yes,
    a) What was the dose and duration of steroids used?________________
    b) Reason for starting steroids?_______________________________________

PROFORMA FOR SURGICAL TB

Title of the Research Project: To evaluate the gaps in the diagnosis and treatment in extra-pulmonary tuberculosis patients under National Tuberculosis Elimination Programme (NTEP) guidelines among various multi-specialties: a multicentric observational study.

DIAGNOSIS

- **Diagnosis: Localized TB/Disseminated TB (Surgical)**

Diagnostic modalities:

- Was any imaging of the affected site done? Yes/No. If no, why?________________________
- Was FNAC of affected site done? Yes/No. If no, why?______________________________
- Was FNAC done under the imaging guidance? Yes/No. If no, why?______________________
- What modality was selected for guiding the FNAC?_________________________
- Was the sample sent for gram stain and bacterial culture sensitivity? Yes/No. If no, why?_____________________
- Was the sample sent for cytology? Yes/No. If no, why?__________________________
- Was the sample sent for AFB? Yes/No. If no, why?__________________________
- Did the patient have to pay for it? Yes/No. If yes, how much?________________________
- Was the sample sent for GeneXpert? Yes/No. If no, why?______________________
- Did the patient have to pay for it? Yes/No. If yes, how much?________________________
- Was the sample sent for TB culture? Yes/No. If no, why?______________________
- Did the patient have to pay for it? Yes/No. If yes, how much?________________________
- In inconclusive cases, was biopsy done? Yes/No. If no, why?__________________
- Was HIV testing done? Yes/No. If no, why?_______________________________
- Was chest X-ray done? Yes/No. If no, why?_______________________________
- On what basis was the anti-tubercular drugs started? (Tick the appropriate option)
    - TB culture
    - GeneXpert
    - Histopathology (caseating granulomas)
    - Acid fast bacilli on microscopy
    - Adenosine deaminase
    - Microbiological evidence of TB elsewhere
    - Empirical ATT trial

TREATMENT

Date of starting TB Rx:_____________ Date of completing TB Rx:__________

- Was HRZE used in intensive phase? Yes/No. If no, why?________________________________
- Was any other agent added to the intensive phase apart from HRZE? Yes/No. If yes, why?________________________________
- Was pyridoxine supplementation started? Yes/No. If no, why?________________________________
- Was duration of continuation phase at least 4 months? Yes/No. If no, why?_________________________
- Was the regimen HRE during continuation phase? Yes/No. If no, why?____________________________
- Was the total duration of ATT more than 6 months? Yes/No. If yes, why?___________________________
- Time from first symptom to starting of the ATT _____________________
- Was the patient started on steroids? Yes/No. If yes,
    a) What was the dose and duration of steroids used?_______________
    b) Reason for starting steroids?____________________________________

PROFORMA FOR UROGENITAL TB

Title of the Research Project: To evaluate the gaps in the diagnosis and treatment in extra-pulmonary tuberculosis patients under National Tuberculosis Elimination Programme (NTEP) guidelines among various multi-specialties: a multicentric observational study.

DIAGNOSIS

- **Diagnosis: Urinary TB/ Male genital tract TB**
- **Extent: Localized TB/Disseminated TB**

Diagnostic modalities:

- Was renal function test done? Yes/No. If no, why?__________________________
- In patients with deranged renal functions, was an urgent ultrasonography of urinary tract done? Yes/No. If no, why?_________________________
- Was urine for routine microscopy sent? Yes/No. If no, why?_____________________
- Was urine for bacterial culture/sensitivity sent? Yes/No. If no, why?_____________________
- Was USG KUB/pelvis done? Yes/No. If no, why?______________________________
- Was urine sent for AFB and TB culture? Yes /No. If no, why?___________________
- Was urine sent for Genexpert? Yes /No. If no, why?___________________
- Did the patient have to pay for the test? Yes/No. If yes, how much?_______________________
- Was early morning urine sample chosen for the tubercular testing? Yes/No. If no, why?___________________________
- How many samples were sent for tubercular testing before declaring that there is no microbiological evidence of TB?____
- Was CECT urography done? Yes/No. Why?__________________________________
- Was intravenous urography done? Yes/No. Why?______________________________
- Was MR urography done? Yes/No. Why?__________________________________
- Was FNAC done from an identifiable collection on imaging? Yes/No. If no, why?_________________________________
- Was it done under imaging guidance? Yes/No. If no, why?______________________
- What was the imaging modality used for guiding the FNAC?_____________________
- In inconclusive cases, was biopsy attempted? Yes/No. If no, why?________________________
- Was chest x-ray done? Yes/No. If no, why?_________________________________
- Was HIV testing done? Yes/No. If no, why?_________________________________
- On what basis was the anti-tubercular drugs started? (Tick the appropriate option)
    - TB culture
    - GeneXpert
    - Histopathology (caseating granulomas)
    - Acid fast bacilli on microscopy

- Adenosine deaminase
- Microbiological evidence of TB elsewhere
- Empirical ATT trial

TREATMENT

Date of starting TB Rx:________________ Date of completing TB Rx:_____________

- Was HRZE used in intensive phase? Yes/No. If no, why?__________________________________
- Was any other agent added to the intensive phase apart from HRZE? Yes/No. If yes, why?__________________________________
- Was pyridoxine supplementation started? Yes/No. If no, why?__________________________________
- Was the regimen HRE during continuation phase? Yes/No. If no, why?_____________________________
- Was the total duration of ATT more than 6 months? Yes/No. If no, why?____________________________
- Time from onset of first symptom to starting of the ATT ______________________
- Was the patient started on steroids? Yes/No. If yes,
  a) What was the dose and duration of steroids used?________________
  b) Reason for starting steroids?_______________________________________
